# Effect of Protease‐Treated Royal Jelly Extract on Facial Wrinkles: A Placebo‐Controlled, Double‐Blind, Parallel‐Group Study

**DOI:** 10.1111/jocd.70503

**Published:** 2025-10-24

**Authors:** Shiho Ikegami, Takashi Ito, Hideto Okamoto, Chizuru Fujikura, Hayate Itatani, Masayuki Yagi, Akio Ohkuma, Nobuaki Okumura, Ayanori Yamaki, Norihiro Shigematsu, Kayo Kunimoto, Masatoshi Jinnin

**Affiliations:** ^1^ Institute for Bee Products and Health Science, R&D Department Yamada Bee Company Inc. Okayama Japan; ^2^ Yamada Bee Company Group Institute for Beauty Science, R&D Department Yamada Bee Company Inc. Tokyo Japan; ^3^ Research Center for Immunological Analysis Inc. Okayama Japan; ^4^ R&D Department Yamada Bee Company Inc. Okayama Japan; ^5^ Department of Dermatology Wakayama Medical University Wakayama Japan

**Keywords:** anti‐wrinkle, dermal thickness, royal jelly, stem cells, water content of the stratum corneum

## Abstract

**Background:**

No clinical trials have reported on the wrinkle‐improving effects of cosmetics which activate stem cells in vitro.

**Aims:**

To evaluate the anti‐wrinkle effects of protease‐treated royal jelly (pRJ)‐containing cream application to the skin.

**Materials & Methods:**

Seventy healthy Japanese women concerned about skin aging were treated with pRJ‐containing cream or placebo for 12 weeks using the split‐face method in a placebo‐controlled, double‐blind, parallel‐group study.

**Results:**

The pRJ‐containing cream group showed an improvement in the maximum depth and average depth of the biggest wrinkle in the crow's feet wrinkle, along with increased water content of the stratum corneum and dermal thickness, compared to the placebo group. Significant decreases in the relative amount of bacterial microbiome of the cheeks were observed only in the pRJ‐containing cream group. In the ex vivo study, expression of COL17A1, epidermal stem cell function marker, was significantly up‐regulated with the use of the pRJ‐containing cream compared with the placebo cream.

**Conclusion:**

This is the first study to demonstrate the wrinkle‐improving effects of pRJ. Our results suggest that pRJ not only controls stratum corneum water content, dermal thickness, and bacterial microbiome, but also affects stem cell competition and mesenchymal stem cell activation. pRJ can reduce or eliminate certain skin concerns of women by improving age‐related wrinkles.

## 1 Introduction

There are two major mechanisms underlying wrinkle formation that differ between the epidermis and the dermis. For example, wrinkles are induced by the loss of skin flexibility due to epidermal dryness or the loss of skin elasticity due to the degeneration of dermal extracellular matrix [1, 2].

Royal jelly, a creamy milky white substance that worker bees digest and subsequently decompose using pollen and nectar in the body, is secreted from their hypopharyngeal and large jaw glands. It contains unique components including 10‐hydroxy‐2‐decenoic acid and 10‐hydroxy decanoic acid, proteins, amino acids, vitamins, and minerals. Previous studies have shown that 10‐hydroxy‐2‐decenoic acid promotes production of filaggrin and collagen in vitro [3, 4]. Our clinical study suggested that applying an essence containing royal jelly ethanol extract significantly improves the water content of the stratum corneum on the cheeks compared to placebo [5]. However, its effect on wrinkles has not been verified.

On the other hand, a study involving protease‐treated royal jelly (pRJ) also reported a significant increase in the water content of the stratum corneum compared to placebo [6]. The pRJ was royal jelly treated with protease and filtered to remove insoluble matter, leaving only the water‐soluble components containing fatty acids, which decomposes proteins and is hypoallergenic. We also previously confirmed that pRJ maintains stem cell properties of epidermal keratinocytes in a three‐dimensional epidermal model [7]. Similarly, pRJ has been found to promote the secretion of collagen‐producing exosomes in adipose‐derived mesenchymal stem cells [8].

Furthermore, healthy skin has higher diversity of bacterial microbiome than skin with psoriasis or atopic dermatitis [9]. Royal jelly contains antibacterial peptides and has been reported to have antibacterial effects on *Staphylococcus aureus* ( *S. aureus* ), *Pseudomonas aeruginosa* , and *Cutibacterium acnes* (*C. acnes*) [10]. However, no studies have reported on the effects of royal jelly application on the skin bacterial microbiome, which might be involved in wrinkle formation.

Therefore, this placebo‐controlled, double‐blind, parallel‐group study firstly aimed to evaluate the impact of pRJ on wrinkles. Skin texture, epidermal turnover, and skin microbiome, which are known to be related to wrinkle formation, were also evaluated. Additionally, focusing on the activation of stem cells in the epidermis and dermis, we measured the water content of the stratum corneum and dermal thickness, which are the expected mechanisms for improving wrinkles and can indirectly reflect stem cell activation. Furthermore, we investigated type XVII collagen (COL17A1) expression ex vivo to confirm whether the results obtained in the clinical trial were associated with the effect of pRJ on epidermal stem cells.

## 2 Materials and Methods

### 2.1 Clinical Trial

#### 2.1.1 Objective

We conducted the present study at the Research Center for Immunological Analysis Inc., between December 2022 and July 2023, recruiting healthy Japanese women aged 40–60 years.

#### 2.1.2 Protocol

The Yamada Bee Company Ethics Committee reviewed and approved the study protocol on December 13, 2022. We conducted the study in accordance with the Declaration of Helsinki (revised at the 64th WMA General Assembly, Fortaleza, Brazil, October 2013) and the Ethical Guidelines for Medical and Biological Research Involving Human Subjects (Ministry of Health, Labour and Welfare, 2022). The study protocol was registered with the University Hospital Medical Information Network (UMIN 000049871). Written informed consent was obtained from the participants after fully explaining the purpose and methods of this study.

#### 2.1.3 Participants

The Research Center for Immunological Analysis Inc. (Okayama, Japan) recruited the participants. The target numbers of participants for the screening, enrollment, and completion of the trial were 120, 70, and 60, respectively. We determined these patient numbers based on similar previous studies involving participants with age‐related wrinkles, where significant difference was observed between the test product and placebo [11]. Exclusion criteria were set based on a previous study [5].

The inclusion criteria were as follows: (1) individuals who suffered from skin aging issues, such as wrinkles, loss of firmness, and sagging skin; (2) healthy Japanese women aged 40–60 years at the time of providing consent; (3) individuals who agreed to comply with regulations during the study; and (4) individuals who fully understood the content of the study and provided written consent.

The exclusion criteria were as follows: (1) individuals who daily used cosmetics similar to the test product; (2) individuals who may exhibit skin allergic symptoms to the test product; (3) individuals who were currently receiving medications prescribed by the dermatologists or who planned to visit dermatologists during the study period (including those who were being treated for atopic dermatitis); (4) individuals with trauma or inflammation at the measurement site; (5) individuals undergoing hormone replacement therapy (e.g., estrogen preparation or progesterone preparations including drinks, patches, and ointments); (6) individuals with experience in esthetic medicine (i.e., hyaluronic acid injection) that affected the measurement site; (7) individuals who were unable to refrain from behavior leading to sunburn, such as visiting tanning salons and outdoor activities, during the study period; (8) individuals who could not avoid beauty care (e.g., epilation or beauty treatment) during the study period after providing consent; (9) individuals who could not maintain a regular lifestyle during the study period; (10) individuals who did not work on a day shift (those who work at night); (11) individuals whose alcohol intake was more than the equivalent of 500 mL beer/day; (12) individuals who had smoked for 3 months before providing consent, including electronic cigarettes; (13) individuals who participated in another clinical trial in the last 3 months; (14) individuals who worked for healthy food companies, cosmetic companies, and pharmaceutical companies; (15) individuals who used cosmetics, quasi‐drugs, drugs, or healthy food products that may affect the study; and (16) individuals who were judged to be unsuitable for enrollment based on other reasons suggested by the principal investigator.

#### 2.1.4 Blinding and Allocation

The test products, a cream containing pRJ (pRJ‐Cr) or placebo cream (PLBO‐Cr), were identical in appearance. We blinded them using symbols “A” or “B,” and delivered to the Research Center for Immunological Analysis Inc. An individual not directly involved in this study conducted the blinding process. Participants were randomly assigned IDs (01–35 and 36–70); participants with IDs 01–35 applied test products A or B to the right or left side of the face, respectively. Meanwhile, IDs 36 to 70 applied test product A to the left side of the face and test product B to the right side. A key opening was conducted after the statistical analysis.

#### 2.1.5 Test Product and How to Use

pRJ‐Cr containing pRJ (Lot. No. 210909), butylene glycol (Kokyu Alcohol Kogyo Co. Ltd.), pentylene glycol (Symrise AG), and phenoxyethanol (Yokkaichi Chemical Co. Ltd.) comprising > 0.018 w/v% 10‐hydroxy‐2‐decenoic acid were prepared by Yamada Bee Company Inc. (Table 1).

**TABLE 1:** Contents of the test product.

pRJ‐Cr
Royal Jelly Extract, Water, Butylene Glycol, Pentylene Glycol, Phenoxyethanol, Caprylic/Capric Triglyceride, Potassium Hydroxide, Acrylates/C10‐30 Alkyl Acrylate Crosspolymer, Carbomer
PLBO‐Cr
Water, Butylene Glycol, Pentylene Glycol, Phenoxyethanol, Caprylic/Capric Triglyceride, Potassium Hydroxide, Acrylates/C10‐30 Alkyl Acrylate Crosspolymer, Carbomer

*Note:* Creams containing protease‐treated royal jelly (pRJ) and placebo are referred to as pRJ‐Cr and PLBO‐Cr, respectively.

The participants applied approximately 1 g (five pushes) of pRJ‐Cr or PLBO‐Cr as the test product to one side of their face as described above, twice a day (morning and evening) for 12 weeks. The other test product was applied to the remaining half of the face under the same conditions. We evaluated the effects of pRJ‐Cr on the skin by comparing with those of PLBO‐Cr. If the participants usually used creams or emollients, they were replaced with the test products. The order of application of the skincare cosmetics was as follows: (1) face wash, (2) regular lotion, (3) regular essence or serum, and (4) test products. The number of days the test products were used and the amount used were recorded based on a diary and the remaining amount.

#### 2.1.6 Endpoints

The primary endpoint was the objective evaluation of the crow's feet wrinkles by measuring the maximum depth of the biggest wrinkle. Secondary endpoints were the objective evaluation of the crow's feet wrinkles by measuring the average wrinkle depth, average depth of the biggest wrinkle, wrinkle volume, % wrinkle area, % skin texture, water content of the stratum corneum, dermal thickness, stratum corneum condition obtained by tape stripping, skin bacterial microbiome, and adverse events. We also performed photographic evaluation of the crow's feet wrinkles to confirm the participant's background at the time of enrolment.

Participants were instructed to remove their facial makeup and to wash their faces using soap without moisturizing ingredients. Subsequently, they acclimatized for 15 min in a room with constant temperature (21°C ± 1°C) and humidity (50% ± 5%) before measurement.

##### 2.1.6.1 Objective Evaluation of the Crow's Feet Wrinkles

Based on the previous study of wrinkles [11], with the participant's eyes lightly closed, the skin analysis program SKINCAST (manufactured by RSI) was applied to the creases at the corners of the eyes, generating a replica of the skin. Wrinkles were aligned using bundled software to evaluate the same part of the replica on each observation day. We three‐dimensionally analyzed the replica using PRIMOS CR (Canfield Scientific Inc., Parsippany, NJ) to measure the following five items [11]:

- Maximum depth of the biggest wrinkle: The maximum depth of the biggest wrinkle within the analyzed area.
- Average wrinkle depth: The average depth of all wrinkles within the analyzed area.
- Average depth of the biggest wrinkle: The average depth of the biggest wrinkle within the analyzed area.
- Wrinkle volume: The sum of the volumes of all wrinkles within the analyzed area.
- % Wrinkle area: The area percentage of wrinkles in the analyzed area.

##### 2.1.6.2 Skin Texture

We evaluated the skin texture of a replica of the cheek using SKINCAST and the reflective replica analysis system ASA‐03RXD (Asch Japan Co. Ltd.) for the following seven items, as reported previously [12]:

- % Texture volume: The volume percentage of skin texture within the analyzed area. ΣW'D′/XY/100 (W′: Width of the groove determined as texture [μm], D′: Depth of the groove determined as texture [μm], X: 4 Width of the analyzed area [mm], Y: Number of lines)
- Average texture depth: The average depth of skin grooves within the analyzed area.
- Number of textures: The number of skin textures within the analyzed area.
  N′/XY (N′: Average number of textures)
- % Volume: The volume percentage of all skin grooves within the analyzed area.
  ΣWD/XY/100 (W: Width of all grooves [μm], D: Depth of all grooves [μm])
- % Texture area: The skin texture area percentage within the analyzed area.
  ΣW'/XY/100
- Maximum depth texture: The maximum depth of skin grooves in the texture within the analyzed area.
- Maximum width texture: The maximum width of skin texture within the analyzed area.

##### 2.1.6.3 Water Content of the Stratum Corneum

We used the Corneometer CM825 (Courage+Khazaka Electronic GmbH) to measure the moisture content of the stratum corneum, according to the previous study [11]. The measurement areas were the left and right cheeks (the intersection of the outer corner of the eye and nose). We calculated the average values by measuring five times and adopting three measured values, excluding the maximum and minimum values.

##### 2.1.6.4 Dermal Thickness

We used the DermaLab (Cortex Technology) to evaluate the average dermal thickness (μm), by measuring three times using ultrasound at the same location on the cheek [13].

##### 2.1.6.5 Stratum Corneum Condition Obtained by Tape Stripping

We collected the corneum layer of the cheek using corneum checker (Asch Japan Co. Ltd.), and imaged the BG‐stained corneocytes by an optical microscope (ECLIPSE E600W; Nikon Corporation), based on the previous similar research [14]. Thereafter, the projected area of the corneocyte, multi‐layer peeling rate, and % nucleated cell were measured using WinRoof V5.6 (Mitani Corporation).

##### 2.1.6.6 Skin Bacterial Microbiome

We used World Fusion's S‐KIN kit, which consists of a swab soaked in physiological saline, to collect resident bacteria on the cheeks. The 16S rRNA V2‐3‐4‐6‐7‐8‐9 region sequences in the obtained DNA sample were amplified by Ion 16S Metagenomics Kit [15] and read by ION S5 sequencer. Low‐quality sequences and short reads (≤ 120 bp) were eliminated from analysis. The bacterial species were identified through 16S rRNA metagenomic analysis. We calculated diversity index (Shannon entropy and Simpson's index, representing alpha diversity) and relative amount as described previously [16].

### 2.2 Ex Vivo Study

#### 2.2.1 Human ex vivo Skin Model

We purchased abdominal skin tissues of a healthy 39‐year‐old female donor with an 11‐mm diameter (NativeSkin©) from Genoskin (Toulouse, France) together with culture medium. Skin explants were cultured with 1 mL medium in a humidified incubator at 37°C with 5% CO_2_ [17]. We topically applied pRJ‐Cr or PLBO‐Cr (20 μL) onto the skin surface of area pre‐defined with a silicon ring twice daily for 24 h. Subsequently, tissues were harvested for immunofluorescence staining.

#### 2.2.2 Histological Analysis

Histological analysis was performed according to the previous studies [18, 19]. Briefly, skin tissues were fixed with a cold 4% paraformaldehyde solution (Nacalai Tesque, Kyoto, Japan) overnight, and ethanol was replaced with G‐NOX (GenoStaff, Tokyo, Japan). We analyzed the expression of COL17A1 and tissue inhibitor of metalloproteinases 1 (TIMP1) using antibodies against COL17A1 (GT1273; rabbit mAb, geneTex, CA, USA) and TIMP1 (#8946; rabbit mAb, Cell Signaling, MA, USA), respectively. Briefly, sections (7‐μm thickness) were treated with 0.3% Triton X‐100 and 1% normal goat serum at room temperature (20°C ± 2°C) for 1 h. Subsequently, the anti‐COL17A1 primary antibody (1:100) or anti‐TIMP1 primary antibody (1:1000) was incubated with the sections overnight at 4°C. Sections were washed with Tween 20‐phosphate‐buffered saline (T‐PBS) and were incubated with goat anti‐rabbit IgG 555 (1:2000; Thermo Fisher Scientific, Inc., USA) for 1 h at room temperature. After washing with T‐PBS, the sections were stained with DAPI‐Fluoromount‐G (Southern Biotech, Birmingham, AL, USA). We obtained the images with an all‐in‐one fluorescence microscope (BZ‐X800; KEYENCE, Osaka, Japan) equipped with Plan Apochromat 20× objective (NA0.75, MRD00205; Nikon, Tokyo, Japan). Red fluorescence was detected using a TRITC filter (ex:545/25 nm, em:605/70 nm, dichroic:565 nm, OP‐87764; KEYENCE, Osaka, Japan). We prepared three non‐consecutive cross‐sections for each tissue, and five images per section were randomly photographed for analysis. Mean intensity was calculated as described previously [20].

#### 2.2.3 Statistical Analysis

Values are presented as the mean ± standard deviation, unless indicated. Intragroup comparisons were performed using paired *t*‐test, and the significance level was corrected according to the number of tests using the Bonferroni method. Student's *t*‐test was used to compare mean values. We used JMP5.1 (SAS Institute Inc.) for all statistical analyses and GraphPad Prism version 7 (GraphPad Software) for the ex vivo study. All tests were two‐tailed, and statistical significance was considered at a level of 5%.

## 3 Results

### 3.1 Clinical Trial

#### 3.1.1 Target Background

**FIGURE 1:**
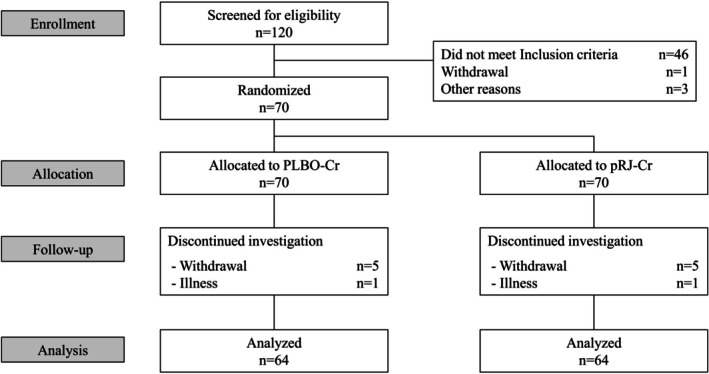
Flow diagram of the double‐blind, split‐face, placebo‐controlled clinical trial. One hundred and twenty participants were screened for eligibility, 70 of whom underwent randomization. After the trial started, 6 participants dropped out. Data of 64 participants who completed the study were analyzed for efficacy and safety. Creams containing protease‐treated royal jelly (pRJ) and placebo are referred to as pRJ‐Cr and PLBO‐Cr, respectively.

A total of 120 female participants underwent the screening test, with 50 found to be ineligible. Among the ineligible participants (*n* = 50), we excluded 46 participants for not meeting inclusion criteria, 1 by withdrawal, and 3 for other reasons. Remaining 70 participants were enrolled, of which 6 participants dropped out (5 by withdrawal and 1 by illness), and 64 participants completed the study. There were no participants with protocol deviations. Figure 1 shows the flow of the analysis, and Table 2 presents the background of the 64 participants at the time of enrollment.

**TABLE 2:** Participant background at the time of enrolment.

Number of participants	64		
Sex	Female		
Age (years) (Min ~ Max)	53.58	±	4.86
	44	~	60

*Note:* Values are mean ± standard deviation unless indicated.

Abbreviations: Max, maximum; Min, minimum.

#### 3.1.2 Analysis Results

##### 3.1.2.1 Primary Endpoint: Wrinkle

**FIGURE 2:**
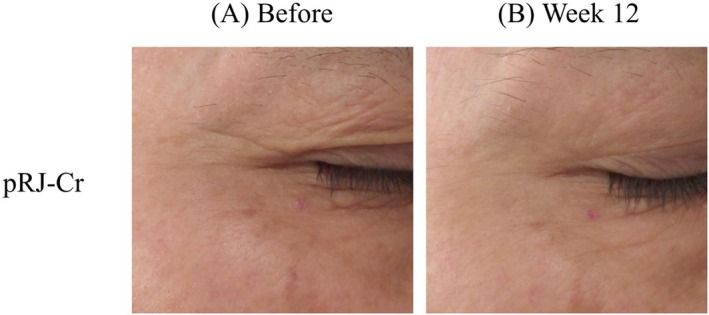
Representative images showing changes in crow's feet wrinkles at baseline (Week 0, A) and after 12 weeks of creams containing protease‐treated royal jelly (pRJ‐Cr) application (Week 12, B). The figure demonstrates a visible reduction in wrinkles due to the application.

The maximum depth of the biggest wrinkle in the pRJ‐Cr group was 133.39 ± 45.47 μm after 6‐week application and 125.39 ± 42.26 μm after 12‐week application, which was significantly reduced compared to the depth at baseline (147.69 ± 43.28 μm, Table 3). Such improvements in the wrinkle depth were not found in the PLBO‐Cr group. Additionally, the % changes in both maximum depth of the biggest wrinkle (86.36% ± 20.41% vs. 104.61% ± 29.43%) and average depth of the biggest wrinkle (96.66% ± 36.88% vs. 112.73% ± 45.23%) at week 12/baseline showed significant improvements in the pRJ‐Cr group compared to those in the PLBO‐Cr group. Wrinkle volume in the pRJ‐Cr group was 1.68 ± 0.95 μm after 6‐week application, which was also significantly reduced compared to baseline (1.89 ± 0.99 μm). The % wrinkle areas in the pRJ‐Cr group were 16.72 ± 0.97 μm after 6‐week application and 16.62 ± 1.12 μm after 12‐week application, which was significantly improved compared to the baseline (18.34 ± 0.95 μm). However, this improvement was also seen in the PLBO‐Cr group. Figure 2 illustrates the representative images showing visible reduction in crow's feet wrinkles after applying pRJ‐Cr.

**TABLE 3:** Change in parameters of the crow's feet wrinkle.

		Baseline	Week 6	Week 12	% Change	
					Week 6/baseline	Week 12/baseline
Max. depth of the biggest wrinkle (μm)	pRJ‐Cr	147.69 ± 43.28	133.39 ± 45.47**	125.39 ± 42.26**	92.34% ± 25.48%	86.36% ± 20.41%^##^
	PLBO‐Cr	152.66 ± 49.43	145.47 ± 45.80	152.91 ± 43.54	97.83% ± 21.73%	104.61% ± 29.43%
Avg. wrinkle depth (μm)	pRJ‐Cr	31.70 ± 11.04	30.70 ± 12.03	32.16 ± 12.72	100.56% ± 32.32%	105.62% ± 42.70%
	PLBO‐Cr	35.98 ± 11.01	37.78 ± 11.80	39.09 ± 12.23	110.21% ± 36.58%	113.94% ± 36.65%
Avg. depth of the biggest wrinkle (μm)	pRJ‐Cr	35.83 ± 13.06	33.55 ± 15.17	33.72 ± 15.66	98.08% ± 39.91%	96.66% ± 36.88%^#^
	PLBO‐Cr	40.95 ± 13.14	42.06 ± 13.18	43.19 ± 13.78	108.24% ± 36.76%	112.73% ± 45.23%
Wrinkle volume (mm^3^)	pRJ‐Cr	1.89 ± 0.99	1.68 ± 0.95**	1.74 ± 0.97	92.42% ± 31.29%	96.07% ± 40.10%
	PLBO‐Cr	2.47 ± 0.96	2.34 ± 0.93	2.41 ± 0.92	98.87% ± 32.60%	103.63% ± 34.48%
% Wrinkle area	pRJ‐Cr	18.34 ± 0.95	16.72 ± 0.97**	16.62 ± 1.12**	91.27% ± 5.19%	91.27% ± 5.19%
	PLBO‐Cr	18.55 ± 0.52	16.64 ± 0.88**	16.83 ± 1.04**	89.68% ± 4.43%	90.72% ± 5.24%

*Note:* Values are presented as mean ± standard deviation (SD). The values between the two groups were compared using Student's *t*‐test. Differences between the baseline, week 6 and week 12 were investigated with paired *t*‐tests using the Bonferroni method. *n* = 64, **p < 0.005 by paired *t*‐test (vs. baseline); #*p* < 0.05, ##*p* < 0.01 by Student's *t*‐test (vs. PLBO‐Cr). Creams containing protease‐treated royal jelly (pRJ) and placebo are referred to as pRJ‐Cr and PLBO‐Cr, respectively.

Abbreviations: Avg, average; Max, maximum.

#### 3.1.3 Secondary Endpoints

##### 3.1.3.1 Water Content of the Stratum Corneum and Dermal Thickness

When the values before and after 12‐week application were compared, the water content of the stratum corneum was maintained in the pRJ‐Cr group (50.07 ± 13.06 vs. 50.69 ± 11.51 A. U.), whereas we observed a significant decrease in the PLBO‐Cr group (49.90 ± 11.69 vs. 42.23 ± 11.75 A. U., Table 4). Moreover, we found a significant difference in the % change in the pRJ‐Cr group compared with the PLBO‐Cr group after 12‐week application (106.37% ± 32.14% vs. 87.35% ± 28.03%). Similarly, the dermal thickness significantly increased in the pRJ‐Cr group (1331.20 ± 194.09 vs. 1366.86 ± 194.05 μm) after 12‐week application, but decreased in the PLBO‐Cr group (1340.26 ± 188.88 vs. 1304.60 ± 164.55 μm, Table 4). Additionally, there was a significant difference in the % change of the pRJ‐Cr group compared to that of the PLBO‐Cr group after 12‐week application (103.10% ± 8.77% vs. 97.86% ± 8.31%).

**TABLE 4:** Changes in the water content of the stratum corneum and dermal thickness.

		Baseline	Week 6	Week 12	% Change	
					Week 6/baseline	Week 12/baseline
The water content of the stratum corneum (A. U.)	pRJ‐Cr	50.07 ± 13.06	46.41 ± 10.76*	50.69 ± 11.51	96.56% ± 24.92%	106.37% ± 32.14%^##^
	PLBO‐Cr	49.90 ± 11.69	45.90 ± 13.30*	42.23 ± 11.75**	94.90% ± 30.62%	87.35% ± 28.03%
Dermal thickness (μm)	pRJ‐Cr	1331.20 ± 194.09	1311.73 ± 198.08	1366.86 ± 194.05*	98.79% ± 8.07%	103.10% ± 8.77%^##^
	PLBO‐Cr	1340.26 ± 188.88	1320.71 ± 187.11	1304.60 ± 164.55*	98.86% ± 8.52%	97.86% ± 8.31%

*Note:* Values are mean ± SD. The values between the two groups were compared using Student's *t*‐test. Differences between the baseline and weeks 6, and week 12 were investigated with paired *t*‐tests using the Bonferroni method. *n* = 64, *p < 0.025, **p < 0.005 by paired *t*‐test (vs. baseline), ##*p* < 0.01 by Student's *t*‐test (vs. PLBO‐Cr). Creams containing protease‐treated royal jelly (pRJ) and placebo are referred to as pRJ‐Cr and PLBO‐Cr, respectively.

##### 3.1.3.2 Skin Texture

**FIGURE 3:**
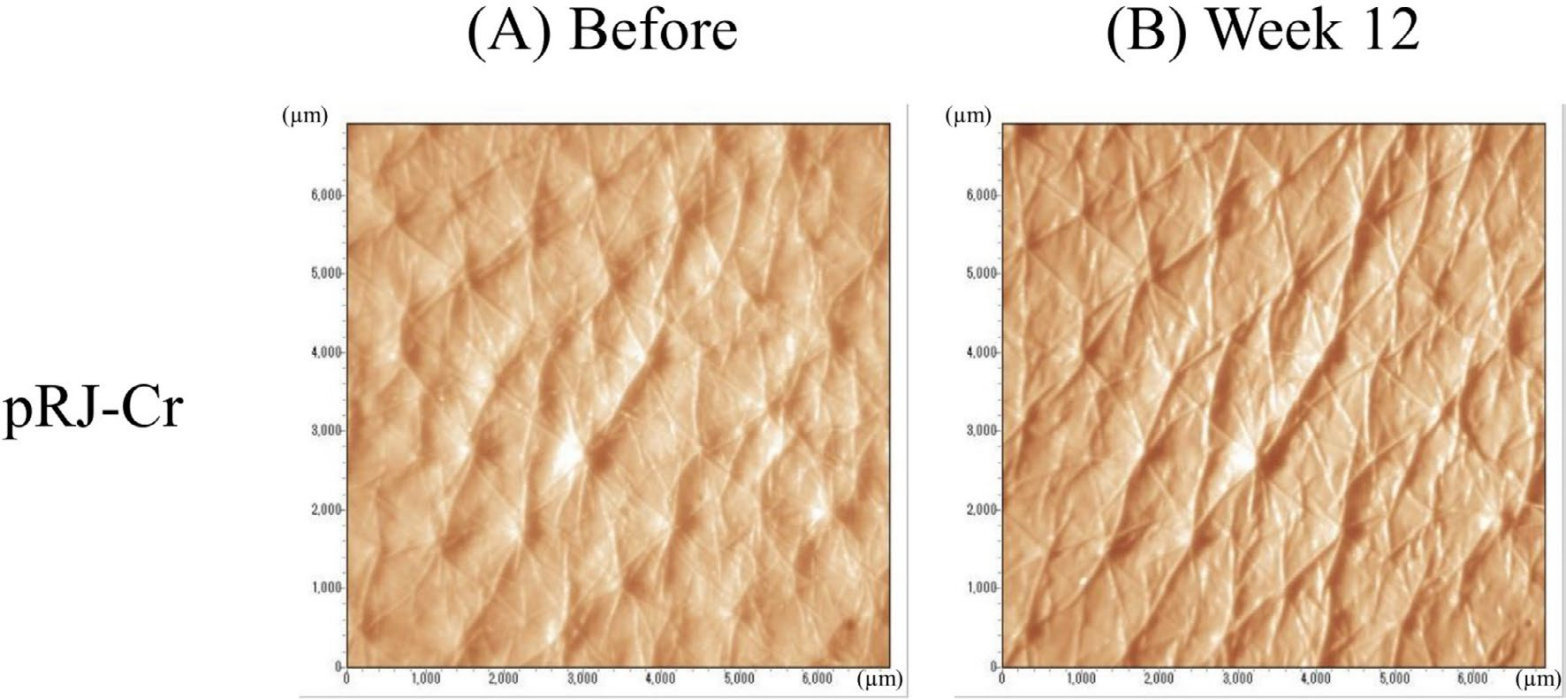
Representative images showing changes in skin texture *at* baseline (Week 0, A) and after 12 weeks of creams containing protease‐treated royal jelly (pRJ‐Cr) application (Week 12, B). The figure demonstrates a visible improvement in skin texture due to the application.

When comparing values before and after the 12‐week application, we found significant improvements in three factors (% texture volume, % texture area, and maximum depth texture) only in the pRJ‐Cr group after the 12‐week application (25.58 ± 11.37 vs. 29.78 ± 8.72, 6.32 ± 2.82 vs. 7.34 ± 2.13, 89.98 ± 0.03 vs. 89.99 ± 0.02, respectively, Table 5). The % volume significantly increased both in the pRJ‐Cr group (120.50 ± 42.82 vs. 135.30 ± 48.27) and PLBO‐Cr group (120.50 ± 50.60 vs. 133.24 ± 53.28) after the 12 weeks application. Average texture depth in the PLBO‐Cr group were 56.70 ± 2.99 μm before application and 57.66 ± 2.57 μm after the 12‐week application, which was significantly increased compared to the values at baseline. Figure 3 illustrates the representative images showing improvements in skin texture after applying pRJ‐Cr.

**TABLE 5:** Changes in the skin texture.

		Baseline	Week 6	Week 12
% Texture volume	pRJ‐Cr	25.58 ± 11.37	30.76 ± 9.56**	29.78 ± 8.72 *
	PLBO‐Cr	25.55 ± 11.83	28.82 ± 9.46	27.25 ± 10.12
Avg. texture depth (μm)	pRJ‐Cr	56.95 ± 2.93	57.65 ± 3.15	57.30 ± 2.92
	PLBO‐Cr	56.70 ± 2.99	57.51 ± 2.60*	57.66 ± 2.57*
Number of textures	pRJ‐Cr	0.78 ± 0.42	0.93 ± 0.38*	0.90 ± 0.33
	PLBO‐Cr	0.79 ± 0.46	0.85 ± 0.39	0.79 ± 0.35
% Volume	pRJ‐Cr	120.50 ± 42.82	133.87 ± 45.38*	135.30 ± 48.27**
	PLBO‐Cr	120.50 ± 50.60	131.94 ± 46.27	133.24 ± 53.28*
% Texture area	pRJ‐Cr	6.32 ± 2.82	7.57 ± 2.39**	7.34 ± 2.13*
	PLBO‐Cr	6.33 ± 2.95	7.09 ± 2.37	6.68 ± 2.49
Max. depth texture (μm)	pRJ‐Cr	89.98 ± 0.03	89.98 ± 0.08	89.99 ± 0.02*
	PLBO‐Cr	89.97 ± 0.07	89.98 ± 0.03	89.98 ± 0.04
Max. width texture (μm)	pRJ‐Cr	354.61 ± 47.96	352.69 ± 49.75	347.40 ± 54.96
	PLBO‐Cr	356.66 ± 55.94	357.26 ± 51.57	349.07 ± 55.53

*Note:* Values are expressed as mean ± SD. % Texture volume is calculated by the formula ΣW'D′/XY/100 (W′: Width of the groove determined as texture [μm], D′: Depth of the groove determined as texture [μm], X: 4 Width of the analysis area [mm], Y: Number of lines). Number of textures is calculated by the formula N′/XY (N′: The average number of textures). % Volume is calculated by the formula ΣWD/XY/100 (W: Width of all grooves [μm], D: Depth of all grooves [μm]). % Texture area is calculated by the formula ΣW'/XY/100. The values between the two groups were compared using Student's *t*‐test. Differences between baseline, week 6, and week 12 were investigated with paired *t*‐tests using the Bonferroni method. *n* = 64, *p < 0.025, **p < 0.005 by paired *t*‐test (vs. baseline). Creams containing protease‐treated royal jelly (pRJ) and placebo are referred to as pRJ‐Cr and PLBO‐Cr, respectively.

Abbreviations: Avg, average; Max, maximum.

##### 3.1.3.3 Stratum Corneum Conditions Obtained by Tape Stripping

We found significant improvements in two factors (% multi‐layer peeling and % nucleated cells) in both groups after 12‐week application (pRJ‐Cr group: 31.51 ± 8.65 vs. 17.57 ± 8.05 and 33.97 ± 14.70 vs. 25.70 ± 10.97, PLBO‐Cr group: 31.29 ± 7.77 vs. 18.25 ± 8.24 and 34.06 ± 16.89 vs. 25.21 ± 11.17, respectively, Table 6).

**TABLE 6:** Changes in the stratum corneum condition obtained by tape stripping.

		Baseline	Week 6	Week 12
Projected area of the corneocyte (μm^2^)	pRJ‐Cr	754.91 ± 79.31	710.39 ± 89.71**	755.43 ± 98.28
	PLBO‐Cr	767.25 ± 99.16	728.90 ± 86.02**	740.61 ± 99.12
% Multi‐layer peeling	pRJ‐Cr	31.51 ± 8.65	13.46 ± 5.76**	17.57 ± 8.05**
	PLBO‐Cr	31.29 ± 7.77	12.39 ± 4.80**	18.25 ± 8.24**
% Nucleated cell	pRJ‐Cr	33.97 ± 14.70	28.23 ± 10.80**	25.70 ± 10.97**
	PLBO‐Cr	34.06 ± 16.89	27.40 ± 12.29**	25.21 ± 11.17**

*Note:* Values are mean ± SD. The values between the two groups were compared using Student's *t*‐test. Differences between baseline, week 6, and week 12 were investigated with paired *t*‐tests using the Bonferroni method. *n* = 64, **p < 0.005, paired *t*‐test (vs. baseline). Creams containing protease‐treated royal jelly (pRJ) and placebo are referred to as pRJ‐Cr and PLBO‐Cr, respectively.

##### 3.1.3.4 Skin Bacterial Microbiome

Both Shannon entropy and Simpson's index tends to have higher indices when the number of species is high and each species is equally present; however, Shannon entropy and Simpson's index are weighted toward rare species and major species, respectively. These parameters significantly increased in the PLBO‐Cr group only after 6‐week application compared to that at baseline (4.70 ± 1.36 vs. 5.44 ± 1.28 and 0.82 ± 0.13 vs. 0.87 ± 0.11, Table 7). Only Shannon entropy was significantly increased in the pRJ‐Cr group (4.64 ± 1.30 vs. 4.95 ± 1.36). Comparing the pRJ‐Cr and PLBO‐Cr groups, Shannon entropy in the PLBO‐Cr group was significantly up‐regulated only at week 6 (4.95 ± 1.36 vs. 5.44 ± 1.28). As to relative amount, significant decreases were observed in *C. acnes*, *Staphylococcus epidermidis* ( *S. epidermidis* ), and *S. aureus* only in the pRJ‐Cr group after 12‐week application (32.39 ± 17.03 vs. 28.27 ± 16.55, 4.26 ± 5.00 vs. 2.33 ± 3.59, and 2.32 ± 2.15 vs. 1.56% ± 1.49%, respectively, Table 8).

**TABLE 7:** Diversity index in three major bacterial species of the skin bacterial microbiome.

		Baseline	Week 6	Week 12
Shannon Entropy	pRJ‐Cr	4.64 ± 1.30	4.95 ± 1.36*	4.91 ± 1.32
	PLBO‐Cr	4.70 ± 1.36	5.44 ± 1.28**^,#^	4.86 ± 1.35
Simpson's Index	pRJ‐Cr	0.82 ± 0.12	0.83 ± 0.12	0.83 ± 0.12
	PLBO‐Cr	0.82 ± 0.13	0.87 ± 0.11**	0.83 ± 0.12

*Note:* Values are expressed as mean ± SD. The values between the two groups were compared using Student's *t*‐test. Differences between baseline, week 6, and week 12 were investigated with paired *t*‐tests using the Bonferroni method. *n* = 64, *p < 0.025, **p < 0.005 by paired *t*‐test (vs. baseline). #*p* < 0.05 by Student's *t*‐test (vs. PLBO‐Cr). Creams containing protease‐treated royal jelly (pRJ) and placebo are referred to as pRJ‐Cr and PLBO‐Cr, respectively.

**TABLE 8:** Changes of relative amount in the skin bacterial microbiome.

The relative amount (%)		Baseline	Week 6	Week 12
*Cutibacterium acnes*	pRJ‐Cr	32.39 ± 17.03	30.00 ± 15.90	28.27 ± 16.55*
	PLBO‐Cr	30.42 ± 18.25	26.00 ± 14.60*	29.19 ± 16.85
*Staphylococcus epidermidis*	pRJ‐Cr	4.26 ± 5.00	2.90 ± 3.60*	2.33 ± 3.59**
	PLBO‐Cr	3.93 ± 3.75	3.00 ± 2.90	2.82 ± 4.84
*Staphylococcus aureus*	pRJ‐Cr	2.32 ± 2.15	1.90 ± 2.20	1.56 ± 1.49**
	PLBO‐Cr	1.87 ± 1.37	2.10 ± 2.40	1.84 ± 2.44

*Note:* Values are expressed as mean ± SD. The values between the two groups were compared using Student's *t*‐test. Differences between baseline, week 6, and week 12 were investigated with paired *t*‐tests using the Bonferroni method. *n* = 64, *p < 0.025, **p < 0.005 by paired *t*‐test (vs. baseline). Creams containing protease‐treated royal jelly (pRJ) and placebo are referred to as pRJ‐Cr and PLBO‐Cr, respectively.

The above results were analyzed using data of participants whose application rate was ≧ 85% based on the number of days on which the test products were applied. When we performed the same analysis on participants who applied appropriate amount (≧ 85%) of the test products based on the remaining amount, in an intragroup comparison before and after application, significant increase was observed in the Shannon entropy only in the pRJ‐Cr group after 12‐week application (Table 9).

**TABLE 9:** Stratified analysis of diversity index in the skin bacterial microbiome.

		Baseline	Week 6	Week 12
Shannon Entropy	pRJ‐Cr	4.55 ± 1.26	4.84 ± 1.40	4.96 ± 1.36*
	PLBO‐Cr	4.77 ± 1.34	5.51 ± 1.22**^,#^	4.85 ± 1.36
Simpson's Index	pRJ‐Cr	0.81 ± 0.13	0.82 ± 0.12	0.83 ± 0.12
	PLBO‐Cr	0.82 ± 0.13	0.88 ± 0.09**^,#^	0.83 ± 0.12

*Note:* Values are expressed as mean ± SD. The values between the two groups were compared using Student's *t*‐test. Differences between baseline, week 6, and week 12 were investigated with paired *t*‐tests using the Bonferroni method. *n* = 48 (RJ‐Cr) and 41 (PLBO‐Cr), *p < 0.025, **p < 0.005 by paired *t*‐test (vs. baseline), #*p* < 0.05 by Student's *t*‐test (vs. PLBO‐Cr). Creams containing protease‐treated royal jelly (pRJ) and placebo are referred to as pRJ‐Cr and PLBO‐Cr, respectively.

#### 3.1.4 Adverse Events

The principal investigator assessed the adverse events using a four‐point scale (“relevant,” “probably relevant,” “probably not relevant,” and “not relevant”) (Table 10). A total of 41 adverse events were reported during the study; all were mild and transient. In six adverse events of pRJ‐Cr group, mainly skin roughness, redness, and irritation, causal relationship with the test products could not be ruled out (“probably relevant”). However, there was no difference in their incidence rate between the pRJ‐Cr and PLBO‐Cr groups. No participant discontinued the study due to adverse effects.

**TABLE 10:** Summary of adverse events.

	pRJ‐Cr		PLBO‐Cr	
	Not relevant	Probably relevant	Not relevant	Probably relevant
Skin roughness	17	1	24	1
Redness	1	1	1	
Irritation	1	3	1	4
Itchiness	4	1	2	

*Note:* Creams containing protease‐treated royal jelly (pRJ) and placebo are referred to as pRJ‐Cr and PLBO‐Cr, respectively. The total number of adverse events was 41: those appeared on both side of faces were counted and listed in both groups.

Five of the six “probably relevant” events in pRJ‐Cr group, except for one skin roughness spontaneously disappeared in one day. Remaining event lasted more than two days, but resolved spontaneously without medical intervention, and no long‐term adverse effects were observed. Thus, the 12‐week use of pRJ‐Cr appears well‐tolerated, with a safety profile comparable to that of the placebo.

#### 3.1.5 Stability

The 10‐hydroxy‐2‐decenoic acid content of the remaining pRJ‐Cr after completing the test was at least 90% of its theoretical value, suggesting high stability.

#### 3.1.6 Ex Vivo Study

**FIGURE 4:**
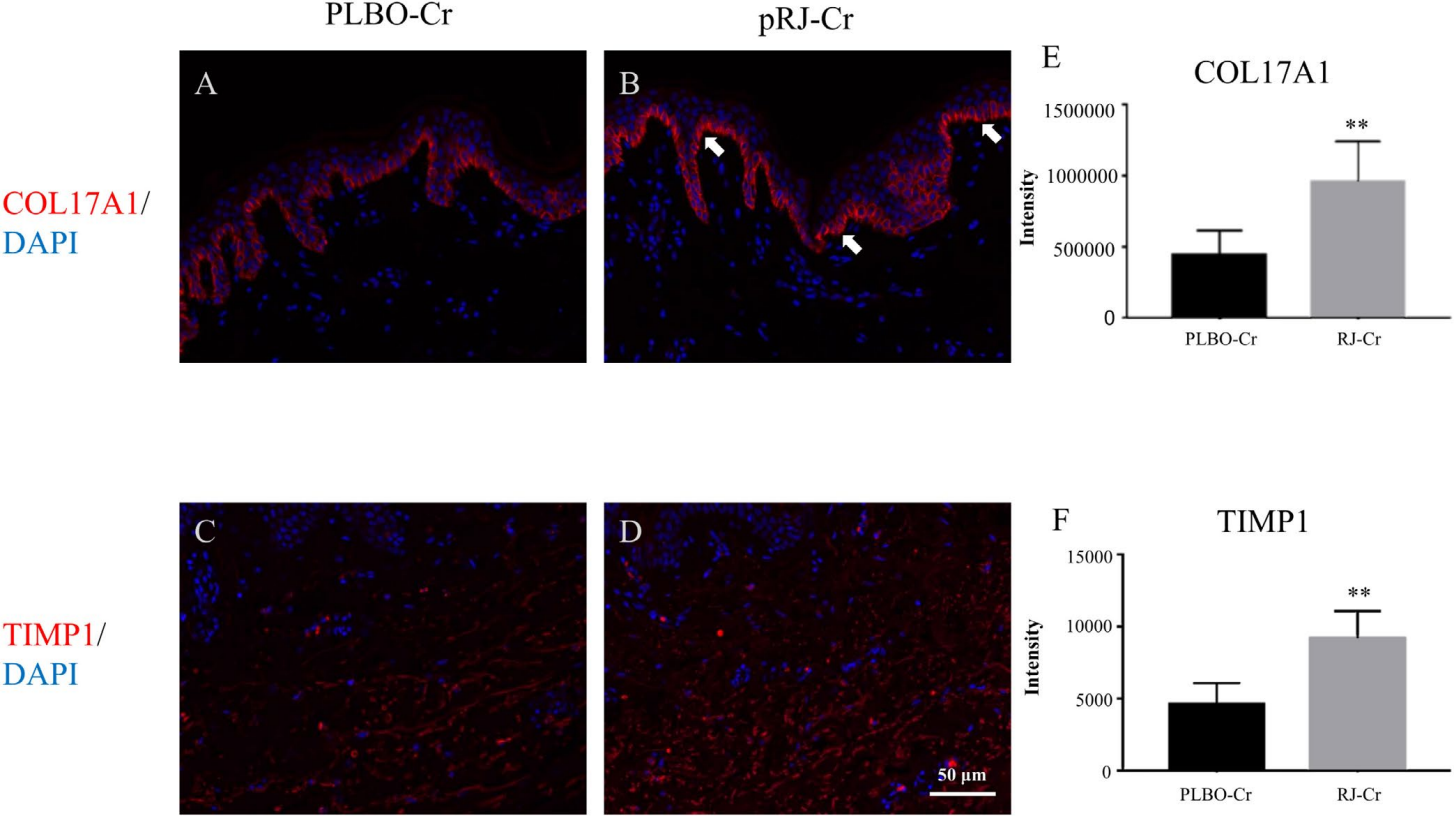
Effect of pRJ‐Cr on the expression of COL17A1 and TIMP1 in a human ex vivo skin model. (A–D) pRJ‐Cr or PLBO‐Cr was topically applied to the skin model twice daily for 24 h. COL17A1 (A and B) and TIMP1 (C and D) were detected by immunofluorescence with specific antibodies (red). Nuclei were visualized using DAPI (blue). Images were obtained and analyzed using BZ‐X800 microscope (Keyence, Tokyo, Japan). pRJ‐Cr application increased the expression of COL17A1 and TIMP1 compared to the PLBO‐Cr application. Three non‐consecutive cross‐sections were prepared for each tissue, and five images per section were randomly photographed for analysis. Representative images were shown. (E, F) Mean intensity was calculated as described in method section. Values are mean ± SD, **p < 0.01 by Student's *t*‐test (vs. PLBO‐Cr). Creams containing protease‐treated royal jelly (pRJ) and placebo are referred to as pRJ‐Cr and PLBO‐Cr, respectively. DAPI, 4′,6‐diamidino‐2‐phenylindole; SD, standard deviation.

Because stem cell competition through COL17A1 is known to diminish with aging [19], leading to the disruption of cell turnover and formation of wrinkles [21], we investigated the expression of COL17A1 in human skin ex vivo to confirm whether the results obtained in the clinical study were attributable to the effect of pRJ on epidermal stem cells. The immunostaining results showed that the pRJ‐Cr application increased the expression of COL17A1 compared to the PLBO‐Cr application (Figure 4A,B,E). COL17A1 was mainly expressed in the epidermal basement membrane, which is consistent with previous reports [22]. TIMP1, which inhibits proteolytic enzyme activity, has been shown to be involved in the inhibition of COL17A1 degradation [19]. pRJ‐Cr application also increased TIMP1 expression in the dermis compared to PLBO‐Cr application (Figure 4C,D,F).

## 4 Discussion

In this study, we examined the anti‐wrinkle effects and other skin benefits of applying pRJ‐containing cream to the face. The pRJ‐Cr group showed an improvement in the maximum depth and average depth of the biggest wrinkle in the crow's feet wrinkle, along with increased water content of the stratum corneum and dermal thickness, compared to the PLBO‐Cr group. Furthermore, the % texture volume, % texture area and maximum depth texture increased after application only in the pRJ‐Cr group. To the best of our knowledge, this is the first study to validate the anti‐wrinkle effects of pRJ in aged individuals with deep wrinkles. We considered parameters with significant differences at week 12 as meaningful. For parameters that exhibited significant differences at both week 6 and week 12 (e.g., maximum depth of the biggest wrinkle), the effect of pRJ was expected to appear at the early stage. On the other hand, although significant differences confirmed only at week 6 (e.g., wrinkle volume or number of textures) may be incidental, other possibility is that prolonged uptake of a single active material may lead to a plateau effect: in spite of an early significant effect, the difference sometimes becomes insignificant after a certain period of time [23, 24], probably due to maintain skin homeostasis. As described in the method section, we performed all measurements under controlled temperature and humidity conditions, and the study period (the end of January to the end of April for all participants) was fixed to minimize seasonal variation. Furthermore, all participants reside in the southern part of Okayama Prefecture, and their work patterns have not changed significantly. Thus, environmental variables are unlikely to affect the results.

The mechanism by which pRJ improve wrinkles seems to be in both the epidermis (increased water content of the stratum corneum) and dermis (increased dermal thickness). Previous reports suggested production of collagen in dermal fibroblasts is induced by royal jelly via mesenchymal stem cell‐derived exosomes and 10‐hydroxy‐2‐decenoic acid [4, 8]. Furthermore, our ex vivo study revealed that the application of pRJ‐Cr increased the expression levels of the epidermal stem cell marker COL17A1 and its regulator TIMP1, indicating that TIMP1 induction by pRJ controls COL17A1 expression, at least partly. Because COL17A1 plays essential roles in stem cell competition [18, 19], pRJ may maintain the number of stem cells, epidermal turnover, and skin homeostasis. Furthermore, pRJ also induces filaggrin via 10‐hydroxy‐2‐decenoic acid [3, 4]. These effects of pRJ may result in increased water content of the stratum corneum.

Furthermore, we firstly demonstrated that the application of pRJ‐Cr significantly reduced the amount of the three types of bacteria in the skin microbiome compared to those before application. Additionally, Shannon entropy significantly increased compared to that before application in participants who applied 85% or more of the test products. This increase may have been due to a decrease in the number of major bacterial species including *C. acnes*, *S. epidermidis* and *S. aureus* . Previous in vitro studies showed that application of royal jelly has antibacterial activity against *C. acnes* and other organisms [10]. Healthy skin has a higher diversity of bacterial microbiome than diseased skin [9]; hence, pRJ may lead to a healthy skin condition.

There are several limitations to this study: first, as our participants were Japanese women aged 44–60, the findings may not be generalizable to other populations. Although statistically significant, the number of samples determined was limited and insufficient to allow statistically based conclusions. In addition, because this study focused on the wrinkle‐improving effect of pRJ, the participants had relatively higher water content in the stratum corneum, and no significant improvement could be observed in intragroup comparisons. Hence, future studies should involve multi‐ethnic cohorts with a wider age range to validate the broader applicability of our results, and should include larger number of participants with lower baseline skin hydration to confirm the moisturizing effect of pRJ.

Second, while there was significant improvement in the maximum depth of the biggest wrinkle and the average depth of the biggest wrinkle in the pRJ‐Cr group, no significant difference was observed in the average wrinkle depth. Average wrinkle depth may not be able to reflect minor changes, and increasing number of participants may be necessary to observe statistical significance. These parameters are common endpoints used in previous studies. Additionally, for example, evaluation targeting changes in the maximum depth of the biggest wrinkle is a practical approach, which is an indicator of concern for participants. However, it has been unknown whether they are the most sensitive indicators of wrinkle change. Other parameters including maximum roughness depth and average roughness may also be included in the future studies. Moreover, it is not yet clear if microbiome changes we demonstrated are causal or correlative to wrinkle improvement from this study. Further *interventional* research (e.g., correlation analyses of longitudinal microbiome monitoring and skin condition during pRJ application) is needed.

Lastly, we conducted ex vivo studies using abdominal skin investigate the mechanisms behind the clinical trial results, based on previous studies [25]. However, facial skin samples were not available, and the effects of pRJ may differ between facial skin and abdominal skin. To demonstrate novel molecular mechanism of wrinkle formation, ex vivo studies of expression of COL17A1 and TIMP1 using facial skin or in vivo experiments using facial skin biopsy specimens would strengthen the conclusion.

In summary, the improvement of maximum depth and average depth of the biggest wrinkle in the crow's feet wrinkle was observed after 12 weeks of application of pRJ‐containing cream in the placebo‐controlled, double‐blind, parallel‐group study. pRJ can reduce or eliminate certain skin concerns of women by improving age‐related wrinkles and skin textures. Our results also suggested that pRJ not only controls stratum corneum water content, dermal thickness, and bacterial microbiome, but also affects stem cell competition and mesenchymal stem cell activation. The limitation of this study included selection of participants, sample size, parameter sensitivity, or mechanistic clarity. Further larger studies, including ex vivo studies using facial skin, will be needed to elucidate the detailed mechanism underlying the effects of pRJ.

## Author Contributions

**Shiho Ikegami:** conceptualization, investigation, project administration, writing – original draft. **Takashi Ito:** investigation. **Hideto Okamoto:** investigation. **Chizuru Fujikura:** supervision, writing – review and editing. **Hayate Itatani:** conceptualization, supervision, writing – review and editing. **Masayuki Yagi:** conceptualization, writing – review and editing. **Akio Ohkuma:** investigation. **Nobuaki Okumura:** writing – review and editing. **Ayanori Yamaki:** conceptualization, supervision, writing – review and editing. **Norihiro Shigematsu:** supervision. **Kayo Kunimoto:** writing – review and editing. **Masatoshi Jinnin:** supervision, writing – review and editing.

## Ethics Statement

The authors confirm that the ethical policies of the journal, as noted on the journal's author guidelines page, have been adhered to and the appropriate ethical review committee approval has been received. This study was reviewed and approved by the Yamada Bee Company Ethics Committee on December 13, 2022 (2022011). The study protocol was registered with the University Hospital Medical Information Network (UMIN 000049871).

## Consent

Fully explaining the purpose and methods of this study to the participants, written informed consent was obtained.

## Conflicts of Interest

Ikegami, Ito, Okamoto, Fujikura, Itatani, Yagi, Okumura, Yamaki, and Shigematsu are employees of Yamada Bee Company, Inc.

## Data Availability

The data that support the findings of this study are available on request from the corresponding author. The data are not publicly available due to privacy or ethical restrictions.

## References

[1] K. D. Agnieszka , From the Characterization of Human Skin to the Development of a Skin Model (ETH Zürich, 2017).

[2] V. Bhardwaj , J. Namkoong , O. Tartar , I. Diaz , J. Mao , and J. Wu , “In Vitro and Ex Vivo Mechanistic Understanding and Clinical Evidence of a Novel Anti‐Wrinkle Technology in Single‐Arm, Monocentric, Open‐Label Observational Studies,” Cosmetics 9 (2022): 80.

[3] L. Gu , H. Zeng , and K. Maeda , “10‐Hydroxy‐2‐Decenoic Acid in Royal Jelly Extract Induced Both Filaggrin and Amino Acid in a Cultured Human Three‐Dimensional Epidermis Model,” Cosmetics 4 (2017): 48.

[4] S. Koya‐Miyata , I. Okamoto , S. Ushio , K. Iwaki , M. Ikeda , and M. Kurimoto , “Identification of a Collagen Production‐Promoting Factor From an Extract of Royal Jelly and Its Possible Mechanism,” Bioscience, Biotechnology, and Biochemistry 68 (2004): 767–773. 15118301 10.1271/bbb.68.767

[5] Y. Maeda , C. Fujikura , T. Asama , et al., “Effect of Facial Application of Essence Containing Royal Jelly Extract on Stratum Corneum Moisture Content: A Placebo‐Controlled, Double‐Blind, Parallel‐Group Study,” Journal of Cosmetic Dermatology 21 (2022): 5747–5754. 35778882 10.1111/jocd.15168

[6] T. Asama , N. Okumura , A. Yamaki , A. Ohkuma , and K. Numano , “Effect of Protease—Digested Royal Jelly Supplementation on Skin Conditions and Safety in Healthy Japanese Adults—A Randomized, Double‐Blind, Placebo‐Controlled, Parallel‐Group Comparison Study,” Japanese Pharmacology & Therapeutics 48 (2020): 79–88.

[7] M. Moriyama , Y. Miyake , N. Okumura , and H. Moriyama , “Royal Jelly Maintains Epidermal Stem Cell Properties by Repressing Senescence,” Biological & Pharmaceutical Bulletin 47 (2024): 2041–2049. 39675970 10.1248/bpb.b24-00607

[8] T. Itoh , T. Degawa , Y. Ito , Y. Akao , and N. Okumura , “Role of Royal Jelly Treated Adipose‐Derived Stem Cell‐Extracellular Vesicles on Fibroblast Proliferation, Migration, and Collagen Production,” Dermatologic Therapy 2023 (2023): 1–21.

[9] C. Callewaert , T. Nakatsuji , R. Knight , et al., “IL‐4Rα Blockade by Dupilumab Decreases *Staphylococcus aureus* Colonization and Increases Microbial Diversity in Atopic Dermatitis,” Journal of Investigative Dermatology 140, no. 1 (2020): 191–202. 31252032 10.1016/j.jid.2019.05.024 PMC7163930

[10] V. Uthaibutra , T. Kaewkod , P. Prapawilai , H. Pandith , and Y. Tragoolpua , “Inhibition of Skin Pathogenic Bacteria, Antioxidant and Anti‐Inflammatory Activity of Royal Jelly From Northern Thailand,” Molecules 28, no. 3 (2023): 996. 36770665 10.3390/molecules28030996 PMC9920569

[11] H. Hattori , N. Tominaga , S. Mugikura , et al., “Evaluation of the Antiwrinkle Effect of a Retinol‐Containing Cream: Randomized, Placebo‐Controlled, Double‐Blind, Right‐Left Comparison Study,” Journal of Japanese Cosmetic Science Society 32 (2008): 297–305.

[12] K. Tominaga , N. Hongo , M. Karato , and E. Yamashita , “Cosmetic Benefits of Astaxanthin on Humans Subjects,” Acta Biochimica Polonica 59, no. 1 (2012): 43–47. 22428137

[13] T. F. Hsu , Z. R. Su , Y. H. Hsieh , et al., “Oral Hyaluronan Relieves Wrinkles and Improves Dry Skin: A 12‐Week Double‐Blinded Placebo‐Controlled Study,” Nutrients 13 (2021): 2220. 34203487 10.3390/nu13072220 PMC8308347

[14] M. Doi , Y. Sagawa , S. Momose , et al., “Topical Treatment With Sacran, a Sulfated Polysaccharide From *Aphanothece sacrum*, Improves Corneocyte‐Derived Parameters,” Journal of Dermatology 44, no. 12 (2017): 1360–1367. 28691388 10.1111/1346-8138.13970

[15] J. Duan , M. Yokota , and Y. Eda , “The Effect of Alpha‐Glucan Oligosaccharide on Facial Skin Flora,” Journal of the Society of Cosmetic Chemists of Japan 55, no. 2 (2021): 176–181.

[16] C. I. R. Casado and A. Monleón‐Getino , “A New R Library for Discriminating Groups Based on Abundance Profile and Biodiversity in Microbiome Metagenomic Matrices,” International Journal of Scientific and Engineering Research 7, no. 10 (2016): 243–253.

[17] Y. Shirasugi , T. Shibata , S. Koike , et al., “Potassium 4‐Methoxysalicylate (4MSK) Exerts a Skin Lightening Effect by Acting on Melanocytes and Keratinocytes,” Journal of Cosmetic Dermatology 24 (2025): e70112. 40071590 10.1111/jocd.70112 PMC11898116

[18] N. Liu , H. Matsumura , T. Kato , et al., “Stem Cell Competition Orchestrates Skin Homeostasis and Ageing,” Nature 568 (2019): 344–350. 30944469 10.1038/s41586-019-1085-7

[19] D. Nanba , F. Toki , K. Asakawa , et al., “EGFR‐Mediated Epidermal Stem Cell Motility Drives Skin Regeneration Through COL17A1 Proteolysis,” Journal of Cell Biology 220 (2021): e202012073. 34550317 10.1083/jcb.202012073 PMC8563287

[20] Y. Kamata , R. Kato , M. Tominaga , et al., “Identification of Keratinocyte Cytoprotectants Against Toxicity by the Multikinase Inhibitor Sorafenib Using Drug Repositioning,” JID Innovations 4, no. 3 (2024): 100271. 38585194 10.1016/j.xjidi.2024.100271 PMC10990978

[21] S. Verdier‐Sévrain and F. Bonté , “Skin Hydration: A Review on Its Molecular Mechanisms,” Journal of Cosmetic Dermatology 6 (2007): 75–82. 17524122 10.1111/j.1473-2165.2007.00300.x

[22] Y. Xiang , Y. Liu , Y. Yang , et al., “Reduced Expression of Collagen 17A1 in Naturally Aged, Photoaged, and UV‐Irradiated Human Skin In Vivo: Potential Links to Epidermal Aging,” Journal of Cell Communication and Signaling 16 (2022): 421–432. 35060094 10.1007/s12079-021-00654-y PMC9411357

[23] R. S. Schwartz and J. Park , “Ingestion of BioCell Collagen, a Novel Hydrolyzed Chicken Sternal Cartilage Extract; Enhanced Blood Microcirculation and Reduced Facial Aging Signs,” Clinical Interventions in Aging 7 (2012): 267–273. 22956862 10.2147/CIA.S32836 PMC3426261

[24] M. Oe , S. Sakai , H. Yoshida , et al., “Oral Hyaluronan Relieves Wrinkles: A Double‐Blinded, Placebo‐Controlled Study Over a 12‐Week Period,” Clinical, Cosmetic and Investigational Dermatology 10 (2017): 267–273. 28761365 10.2147/CCID.S141845 PMC5522662

[25] J. Sekyoo , Y. Seokjeong , K. Sungwoo , et al., “Anti‐Wrinkle Benefits of Peptides Complex Stimulating Skin Basement Membrane Proteins Expression,” International Journal of Molecular Sciences 21 (2020): 73. 10.3390/ijms21010073 PMC6981886 31861912

